# Absent Testis With a Mediastinal Germ Cell Tumor

**DOI:** 10.7759/cureus.24472

**Published:** 2022-04-25

**Authors:** Shreyas Bellur, Sreekar Balasundaram, Ashitha Nanaiah

**Affiliations:** 1 Department of Cardiothoracic Surgery, St. John’s Medical College Hospital, Bangalore, IND; 2 Department of Pathology, St. John's Medical College Hospital, Bangalore, IND

**Keywords:** chemotherapy, surgical exicision, absent testis, mixed germ cell tumor, mediastinal germ cell tumor

## Abstract

Primary mediastinal mixed germ cell tumors (PMMGCTs) are rare, aggressive tumors that, at diagnosis, are typically metastatic. A 22-year-old male with a three-month history of cough, chest pain, and fever presented to our outpatient department. Clinical examination showed reduced left-sided air entry in the left hemithorax, with a non-palpable left testis. Imaging suggested a large anterior mediastinal mass and an absent left testis. Multiple biopsies revealed only necrotic tissue, and laboratory investigations showed elevated alpha-fetoprotein levels. A provisional diagnosis of mediastinal germ tumor was made, and surgical excision was planned given absent nodal or distant metastasis. Intraoperatively, a densely adherent bosselated mass was found. A biopsy revealed a mixed germ cell tumor with a predominant seminoma component and chemotherapy with cisplatin and ifosfamide was advised. However, the patient was lost to follow-up after one cycle. PMMGCTs possibly occur due to reverse migration. These tumors warrant an early diagnosis due to their highly aggressive nature. A multimodal approach with chemotherapy with surgical resection is recommended. Our case sheds light on the possible mechanism and emphasizes the impact of early diagnosis.

## Introduction

Primary mediastinal germ cell tumors are rare, aggressive neoplasms usually diagnosed in young adult males [1]. Based on their histology, they are classified into seminomatous and non-seminomatous germ cell tumors [2]. Mixed germ cell tumors form a rare subset of non-seminomatous germ cell tumors and are considered ‘poor risk’ tumors [2]. We present an interesting case of a primary mediastinal mixed germ cell tumor in a patient with an absent testis.

## Case presentation

A 22-year-old male with a three-month history of cough, chest pain, and intermittent fever presented to our center. His sputum was clear, mucoid, and non-foul smelling, with dull and continuous chest pain with no variation with exertion or posture. He reported no prior episodes of similar symptoms, hemoptysis, and tuberculosis.

**Figure 1:**
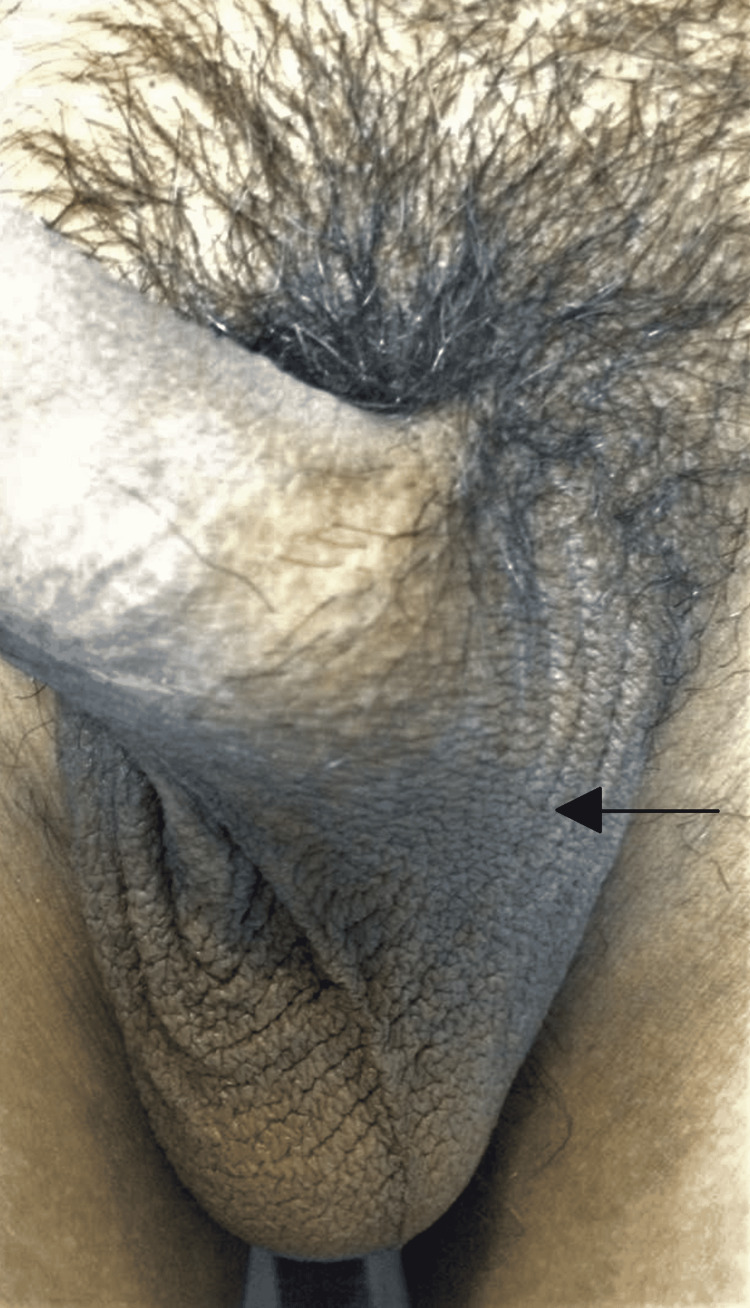
Scrotum showing absent left testis

**Figure 2:**
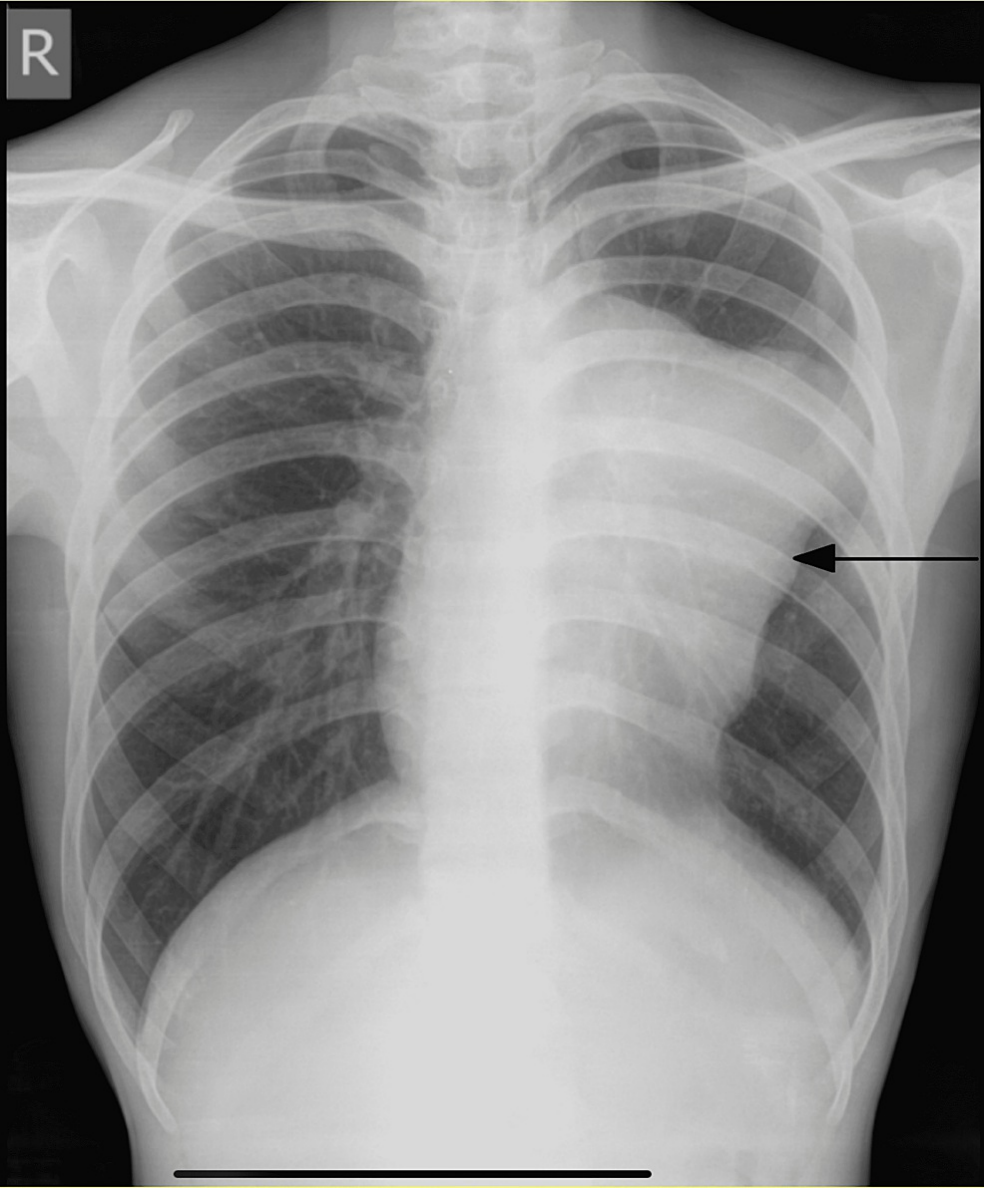
Preoperative chest x-ray showing a left-sided opacity

**Figure 3:**
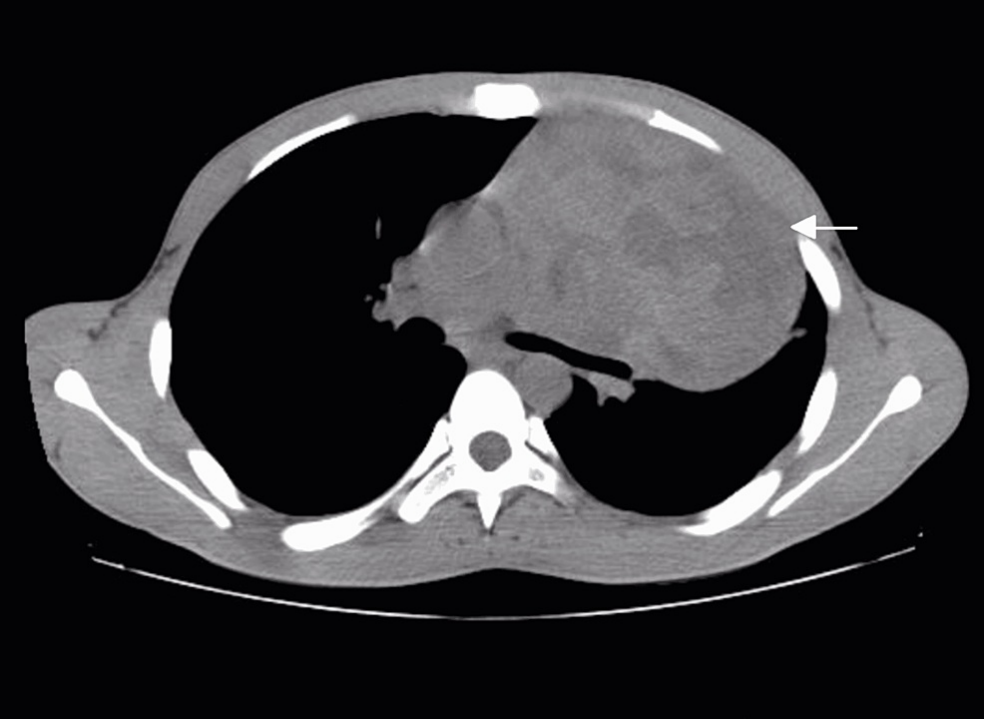
Chest computed tomography showing a well-defined, heterodense anterior mediastinal mass occupying the left hemithorax

The examination was significant for reduced air entry in the left hemithorax and a non-palpable left testis on scrotal examination (Figure 1). There was no evidence of lymphadenopathy. His chest x-ray showed a homogenous opacity occupying the left hemithorax (Figure 2). Computed tomography (CT) of the chest showed a well-defined heterodense anterior mediastinal mass, measuring 12.5 x 10.2 x 12 cm extending to the left hemithorax as well as a compressed left main bronchus (Figure 3). There was no evidence of mediastinal lymphadenopathy. Ultrasound examination of the scrotum and abdomen revealed an absent left testis, right testicular microlithiasis, and calcified granulomas in the liver.

Laboratory investigations showed an elevated level of alpha-fetoprotein (AFP). Beta-human chorionic gonadotropin (beta-HCG), blood count, and metabolic profile were unremarkable.

We ordered a biopsy to establish the diagnosis. Unfortunately, both ultrasound-guided and CT-guided biopsies showed only necrotic and hemorrhagic tissue. After the tumor board discussion, we made a preoperative diagnosis of a germ cell tumor and counseled the patient for surgical excision as there was no evidence of nodal or distant metastasis.

**Figure 4:**
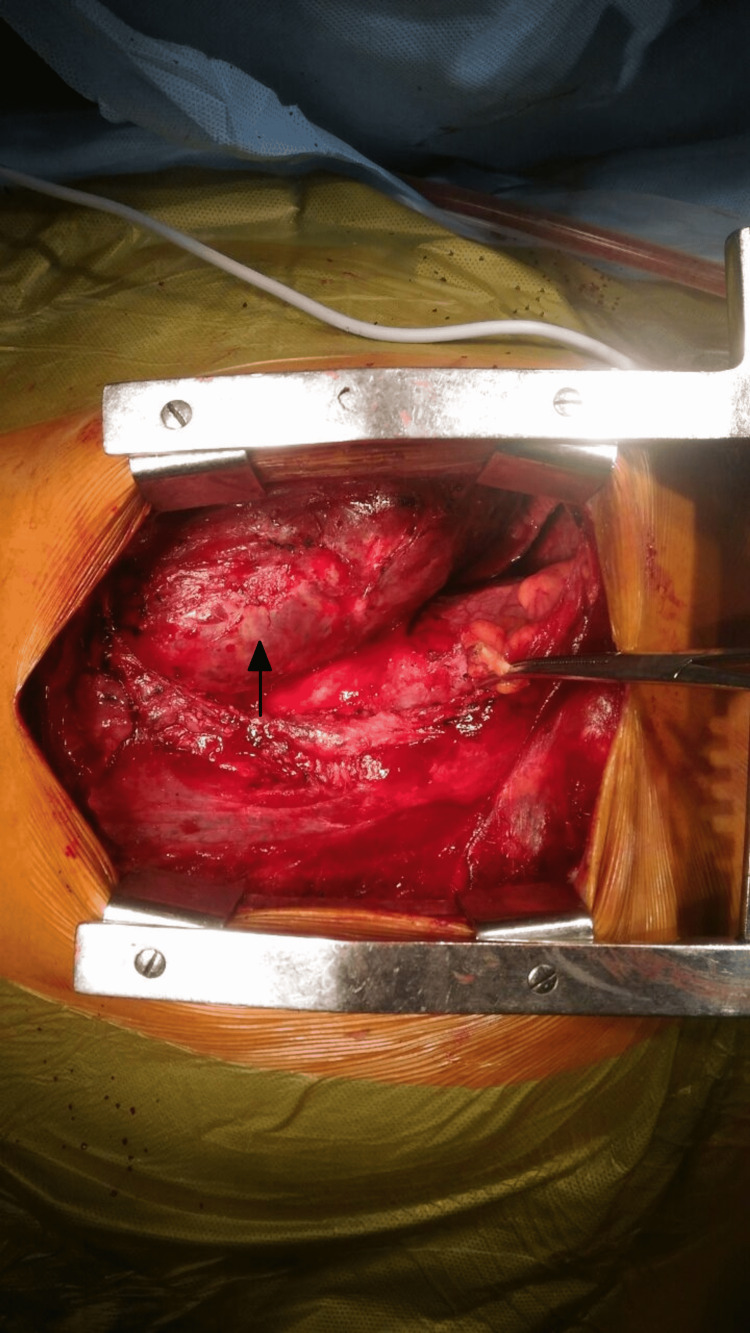
Intraoperative view of the mass

**Figure 5:**
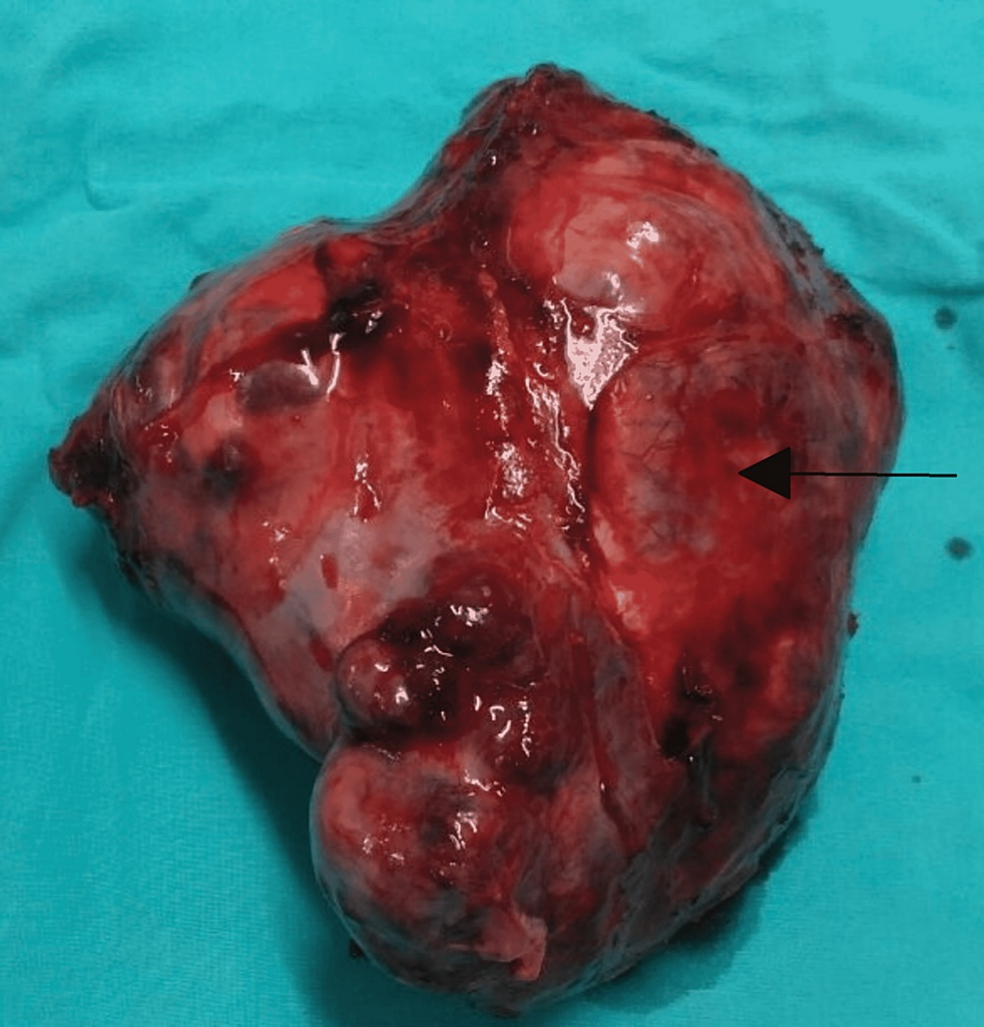
Specimen showing a congested bosselated surface

Intraoperatively, we found a highly vascular mass with a bosselated surface. It measured 17 x 14 x 10 cm and was densely adherent to the lung (apical segment and lingula), chest wall, and mediastinum. The mass demonstrated only solid components and was completely excised along with the apical segment and lingula of the left lung upper lobe, as we suspected local invasion, and was sent for biopsy (Figures 4, 5).

**Figure 6:**
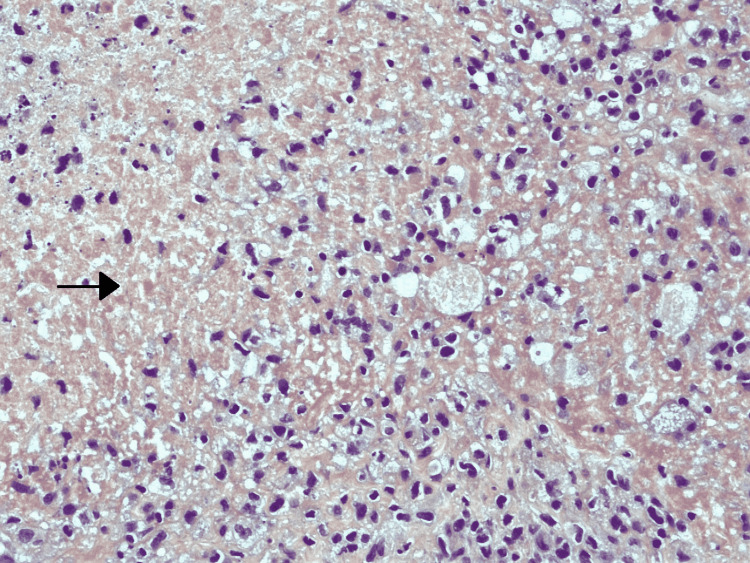
Histopathological specimen showing fibrocollagenous stroma with areas of necrosis The arrow shows necrosis.

**Figure 7:**
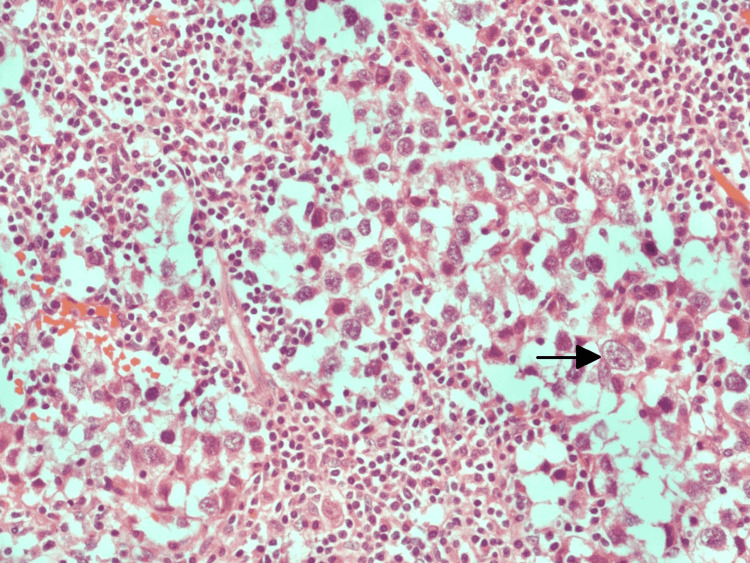
Histopathological specimen showing seminoma The arrow shows a nest of large cells with coarse chromatin and prominent nucleoli.

**Figure 8:**
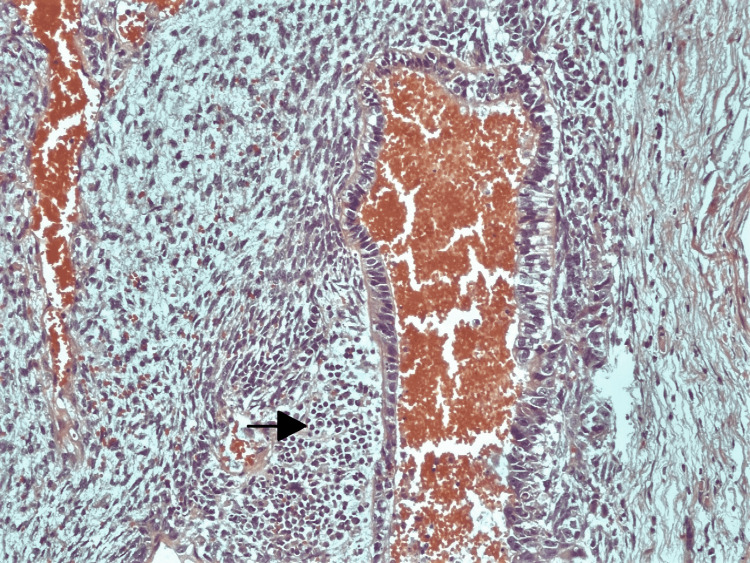
Histopathological specimen showing yolk sac tumor cells The arrow shows tumor cells with clear cytoplasm and clumped chromatin.

**Figure 9:**
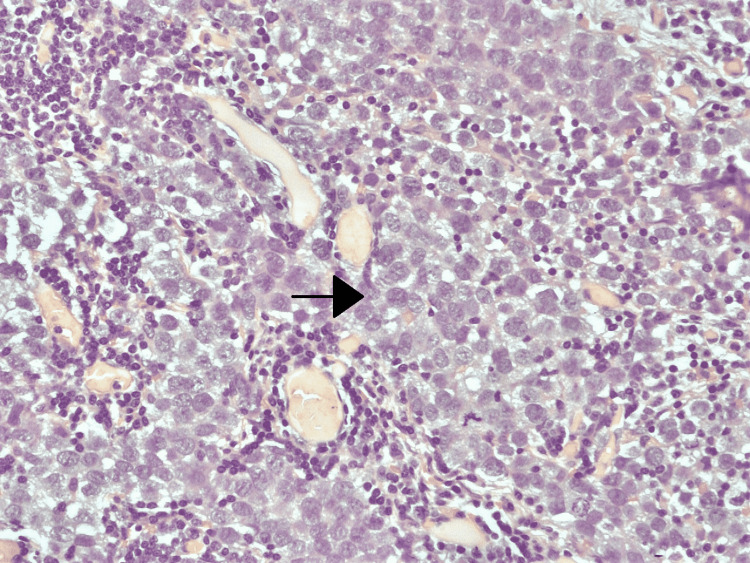
Histopathological specimen showing embroyonal carcinoma The arrow shows large undifferentiated cells in nests and sheets.

**Figure 10:**
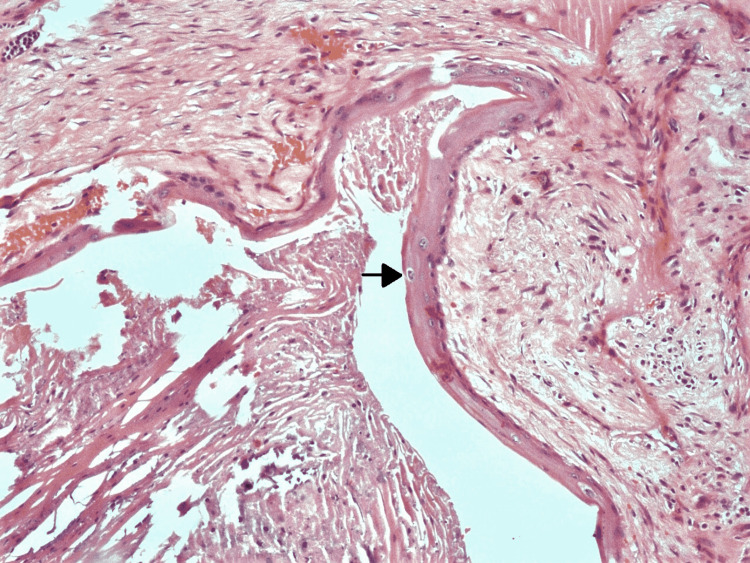
Histopathological specimen showing mature teratoma The arrow shows hyaline cartilage.

The biopsy revealed fibrocollagenous stroma with congested vascular channels (nearly 90% of the tumor). The rest (10%) showed the following components: seminoma (75%), yolk sac tumor (5%), embryonal carcinoma (10%), and teratoma with focal immature neuroepithelial tissue (10%) (Figures 6-10). There was no evidence of lymphovascular, perineural, or local invasion. Immunohistochemistry (IHC) was positive for Sal-like protein 4 (SALL-4, 3+), cluster of differentiation 117 (CD117, 3+), cluster of differentiation 30 (CD30, 1+) and placental-like alkaline phosphatase (PLAP, 1+).

His postoperative period was uneventful with an improved functional status: Eastern Cooperative Oncology Group (ECOG) score of 1 from a preoperative score of 3. The patient was lost to follow-up after receiving one dose of chemotherapy with cisplatin and etoposide.

## Discussion

PMMGCTs comprise a rare subset of non-seminomatous mediastinal germ cell tumors with only a few reported cases. To the best of our knowledge, our case is the first to describe a PMMGCT with an absent ipsilateral testis. The pathogenesis of mediastinal germ cell tumors is not clearly defined. However, one possible mechanism could be reverse migration [3]. This mechanism is supported by the common origin of testicular and mediastinal germ cell tumors. Our case also favors this mechanism as evidenced by the absence of the ipsilateral testis.

At the time of presentation, most PMMGCTs are symptomatic predominantly due to mass effect and tumor necrosis. They are usually big owing to their aggressiveness, with symptoms ranging from low-grade fever, weight loss, and chest pain to superior vena cava syndrome, hoarseness of voice, and lung collapse. Our patient presented similarly with signs of inflammation with intermittent cough.

We suggest estimating the levels of AFP and beta-HCG in all mediastinal masses. In contrast to seminomatous germ cell tumors, PMMGCTs show markedly elevated levels of AFP with a variable elevation in beta-HCG levels [1]. Imaging yields an irregular mass usually with evidence of local invasion, on CT [4]. We suggest an early tissue diagnosis in such cases due to the highly aggressive nature of this neoplasm. As in our case, radiology-guided biopsies may not be feasible, owing to the vascularity and size of the tumor. In such cases, we recommend an open biopsy or even complete surgical excision if there is no evidence of local advancement or metastasis as in our case. It is important to rule out metastasis from testicular germ cell tumors by ultrasonography as palpation is not very reliable [5].

Histopathological examination shows more than one component of germ cell tumors. Our case showed a predominance of the seminomatous component followed by the embryonal carcinoma, immature teratoma, and yolk sac tumor component. IHC is usually positive for seminoma markers CD117, PLAP, embryonal carcinoma marker CD30 and SALL-4 [6,7].

The five-year prognosis is dismal as there is usually evidence of metastasis at the time of presentation [8]. Therefore, treatment is multimodal with chemotherapy with cisplatin, etoposide, and ifosfamide followed by surgery. In our case, as there was no evidence of local or distant metastasis, our tumor board suggested complete surgical excision followed by chemotherapy. We recommend regular monitoring of serum tumor markers to monitor response to therapy and detect any recurrence.

## Conclusions

Primary mediastinal mixed germ cell tumors are highly aggressive neoplasms that usually present with evidence of metastasis at the time of diagnosis. Our case supports the mechanism of reverse migration for its pathology. Early diagnosis is warranted as it significantly increases the chance of survival. A multimodal approach with chemotherapy and surgery is the current recommendation for treatment.
